# Synthesis and photocatalytic activity of mesoporous g-C_3_N_4_/MoS_2_ hybrid catalysts

**DOI:** 10.1098/rsos.180187

**Published:** 2018-05-16

**Authors:** Yirong Qi, Qinghua Liang, Ruitao Lv, Wanci Shen, Feiyu Kang, Zheng-Hong Huang

**Affiliations:** 1 State Key Laboratory of New Ceramics and Fine Processing, Tsinghua University, Beijing 100084, People's Republic of China; 2 Key Laboratory of Advanced Materials (MOE), Tsinghua University, Beijing 100084, People's Republic of China

**Keywords:** photocatalyst, heterojunction, graphitic carbon nitrides

## Abstract

The key to solving environmental and energy issues through photocatalytic technology requires highly efficient, stable and eco-friendly photocatalysts. Graphitic carbon nitride (g-C_3_N_4_) is one of the most promising candidates except for its limited photoactivity. In this work, a facile and scalable one-step method is developed to fabricate an efficient heterostructural g-C_3_N_4_ photocatalyst *in situ* coupled with MoS_2_. The strong coupling effect between the MoS_2_ nanosheets and g-C_3_N_4_ scaffold, numerous mesopores and enlarged specific surface area helped form an effective heterojunction. As such, the photocatalytic activity of the g-C_3_N_4_/MoS_2_ is more than three times higher than that of the pure g-C_3_N_4_ in the degradation of RhB under visible light irradiation. Improvement of g-C_3_N_4_/MoS_2_ photocatalytic performance is mainly ascribed to the effective suppression of the recombination of charge carriers.

## 1. Introduction

Energy issues and environmental pollution are among the most serious challenges for humans in the twenty-first century. Therefore, photocatalytic technology that can use inexhaustible and clean solar energy to solve these problems has attracted great attention. Graphitic carbon nitride (g-C_3_N_4_) is a novel polymeric semiconducting material and was first used as a visible light photocatalyst for the hydrogen reaction in 2009 by Wang *et al*. [1]. Since then, g-C_3_N_4_ has elicited great excitement as an attractive photocatalyst. Compared with traditional photocatalysts, such as TiO_2_, CdS and ZnO, g-C_3_N_4_ is more promising because of the following advantages. firstly, the moderate band gap of approximately 2.7 eV [2], which enables the material to harvest visible light; secondly, the stability, including chemical stability, which means that g-C_3_N_4_ remains almost undissolved in acid, alkali or organic solvents at ambient atmosphere [3,4], and also thermal stability, which means the material only starts to decompose above 600°C [1]; thirdly, the cost-effectiveness and environmental friendliness, because g-C_3_N_4_ is mainly composed of earth-abundant elements (C and N) and can be easily synthesized from cheap precursors like cyanamide [5,6], dicyanamide [7], melamine [8], urea [9] and thiourea [10] via thermal condensation at approximately 550°C.

However, there are still some drawbacks of g-C_3_N_4_ that hinder its further application; in particular, the quick recombination of photo-induced electrons and holes significantly reduces the photocatalytic efficiency. As we know, the key point of a photocatalytic reaction is the reduction and oxidation triggered by photo-induced electrons and holes. Owing to the special π-conjugated structure [11,12], electrons easily combine with holes in the aromatic heterocyclic g-C_3_N_4_. Therefore, there are already various strategies to suppress the recombination of charge carriers, such as textural design to generate mesopores in the bulk g-C_3_N_4_ [13–18], a supramolecular preorganization approach [19–21], exfoliation into thin layers [22,23], elemental doping [24–30], copolymerization [31,32] and nanostructure engineering [33–35].

In addition to the above-mentioned methods, a more efficient solution is to construct a semiconductor–semiconductor heterojunction to enhance the separation efficiency of photo-generated carriers [36–38]. There are already plenty of methods for coupling g-C_3_N_4_ with TiO_2_ [39,40], CdS [41,42], ZnO [43,44], WO_3_ [45,46] etc. to construct a heterojunction. Among these candidate counterparts, MoS_2_ has been attracting tremendous attention owing to its two-dimensional semiconducting properties because a two-dimensional/two-dimensional heterojunction can endow a higher charge mobility. Density functional theory (DFT) calculations have suggested that, after coupling MoS_2_ onto g-C_3_N_4_, electron transfer from MoS_2_ to g-C_3_N_4_ results in an electron-rich region on g-C_3_N_4_ [47]. There have been some studies that have tried to integrate MoS_2_ with g-C_3_N_4_ to construct this kind of heterojunction [47–56], but the preparation process was either too complicated, comprising reduplicate steps of hydrothermal treatment and calcination, or limited by using poisonous reactants like H_2_S.

Here, a facile and scalable one-step synthesis method is proposed to develop g-C_3_N_4_/MoS_2_ hybrid photocatalysts. This one-pot preparation route is very easy and eco-friendly, avoiding harmful reactants. In addition, numerous mesopores, accompanied by a greatly enlarged specific surface area (SSA) of the pristine g-C_3_N_4_, are simultaneously introduced. Most importantly, a better contact interface and strong Mo─N coupling effect formed between g-C_3_N_4_ and MoS_2_ efficiently improves the charge separation, resulting in a highly improved photocatalytic performance.

## 2. Experimental

### 2.1. Synthetic procedures

**Figure 1.**
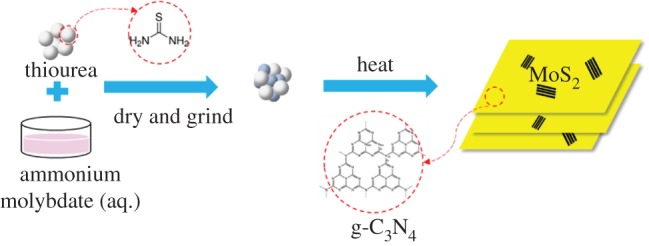
Schematic illustration of the synthesis of g-C_3_N_4_/MoS_2_ hybrid catalysts.

The g-C_3_N_4_/MoS_2_ composite was prepared by a one-pot procedure, which involves directly heating the mixture of thiourea and ammonium molybdate((NH_4_)_6_Mo_7_O_24_·4H_2_O) under the same conditions as shown in figure 1. That is, 0.02 g, 0.05 g and 0.1 g of ammonium molybdate was dissolved in a specified amount of water by magnetic stirring. The solution was slowly dripped into 4 g of thiourea powder. The mixture was then evaporated at 80°C and ground into powder. The samples were named as g-C_3_N_4_/0.5% MoS_2_, g-C_3_N_4_/1.25% MoS_2_ and g-C_3_N_4_/2.5% MoS_2_. Likewise, g-C_3_N_4_ was synthesized by heating 4 g thiourea (CN_2_H_4_S) in a tube furnace at a rate of 2°C min^−1^ to 500°C for 2 h. The sample was cooled down to room temperature and washed with deionized water. All the above-mentioned heating procedures were under an argon flow. All the chemicals used were of analytical grade.

### 2.2. Material characterization

The crystal structures of all the catalysts were measured by powder X-ray diffraction (XRD) with D/max-2500 and a CuK*α* source (*λ* = 0.1541 nm). Fourier transform infrared (FTIR) spectra were recorded by a Perkin-Elmer Spectrum 100 FTIR spectrometer using the KBr pellet technique. The morphology of all the samples was characterized under scanning electron microscopy (SEM; LEO1530) and transmission electron microscopy (TEM; JEOL 2100F, 200 kV). The Brunauer–Emmett–Teller (BET) SSA was calculated from the N_2_ adsorption isotherms obtained using volume adsorption apparatus (autosorb-1) at 77 K. The pore size distributions (PSDs) were determined by DFT. Ultraviolet–visible (UV−vis) diffuse reflection spectroscopy (DRS) was performed on a Hitachi UV-2600 spectrophotometer equipped with an integrating sphere assembly and using BaSO_4_ as a reference. The photoluminescence (PL) spectra were measured on a fluorescence spectrometer (Edinburgh Instruments, FLS920) with an excitation wavelength of 370 nm. The surface chemical composition was measured using an X-ray photoelectron spectroscope (XPS; ESCA 5500MT; PerkinElmer Inc., USA) with an AlK*α* X-ray line (1486.6 eV). The valences of Mo were determined by the deconvolution of Mo3d peaks.

### 2.3. Photocatalytic measurement

The photocatalytic performance of all the samples was evaluated by degrading organic rhodamine B (RhB) dyes in aqueous solution under visible light irradiation using a 500 W Xe lamp with a cut-off filter (l > 400 nm). Specifically, 30 mg of catalyst was suspended in 150 ml of the RhB solution at a concentration of 2 × 10^−5^ mol l^−1^. The suspension was stirred for 120 min in the dark to achieve adsorption equilibrium before the light irradiation. At intervals of 15 min, 2 ml of the solution was collected by a syringe with a microspore filter (220 nm) to remove the photocatalyst. The concentration of RhB was analysed by UV–vis spectrophotometry, according to the maximum light absorption peak at 553 nm. The reaction rate was calculated by *C*/*C*_0_, in which *C*_0_ represents the concentration immediately after dark absorption. The photocatalytic activity of commercial MoS_2_ powder was measured using the same method as a blank control.

The stability of the catalyst was tested by repeated photocatalytic measurements for three cycles. After each cycling test, a sample was collected and washed with distilled water via filtration, then the as-obtained sample was dried at 80°C for 8 h for the next cycle.

### 2.4. Photoelectrochemical measurements

The transient photocurrent measurements were performed in a Bio-Logic electrochemical station with a standard three-electrode system. The working electrodes were prepared as follows: 2 mg powder samples were dispersed in 0.5 ml dimethylformamide with 20 µl Nafion for 30 min under ultrasonic treatment. This suspension was uniformly coated onto a substrate of 1 × 1 cm^2^ fluorine-doped tin oxide (FTO) glass. The electrodes were then dried at 80°C and sintered at 150°C for 2 h to improve adhesion. Before using, FTO glasses were cleaned with ultrasonic washing with acetone, ethanol and distilled water sequentially for 10 min each time, repeating three times. Na_2_SO_4_ (0.5 M) was used as the electrolyte. A Pt flake as a counter electrode, a saturated calomel electrode as a reference, together with an as-prepared working electrode were immersed into the Na_2_SO_4_ aqueous solution to form the three-electrode system. The light source was the same as in the photocatalytic measurement. The photocurrent responses were measured without any bias voltage.

## 3. Results and discussion

**Figure 2.**
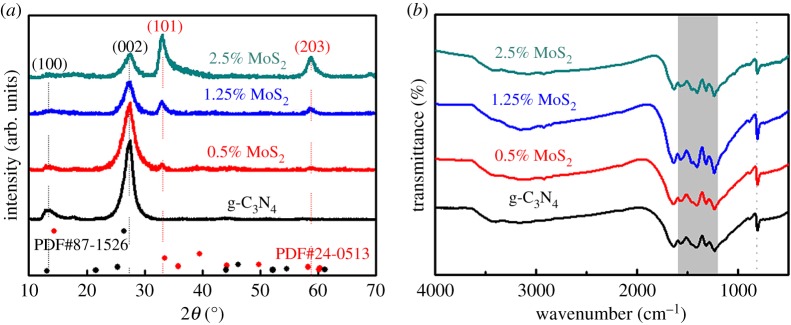
(*a*) XRD patterns and (*b*) FTIR spectra of g-C_3_N_4_, g-C_3_N_4_/0.5% MoS_2_, g-C_3_N_4_/1.25% MoS_2_ and g-C_3_N_4_/2.5% MoS_2._

Existing phases and crystal structures can be confirmed by XRD, as shown in figure 2*a*. The typical peaks of molybdenite 2H1 were detected at approximately 33° and 58°, which can be assigned to the (101) and (110) crystal planes of MoS_2_ (JCPDS#24-0513), respectively. This result reveals the high crystal quality of the as-prepared MoS_2_ layers. The relative peak intensities of these two to the neighbouring peak at 27° increased with the amount of MoS_2_. The two distinct peaks of pristine g-C_3_N_4_ at approximately 13° and 27° are identified as the (100) and (002) crystal planes of graphitic structures (JCPDS#87-1526), corresponding to the in-plane trigonal nitrogen repeated units and periodic stacking layers [34], respectively. The intensity of the g-C_3_N_4_ peak (002) decreased with the increase of MoS_2_, indicating that the MoS_2_ formed was mostly lying in the g-C_3_N_4_ (002) parallel direction. Moreover, the g-C_3_N_4_ phase is also identified by the characteristic FTIR peaks at 810 cm^−1^ and 1200–1600 cm^−1^, shown in figure 2*b*, corresponding to the breathing mode of the triazine units and stretching modes of the CN heterocycles, respectively. Both the XRD and FTIR results confirm the combination of g-C_3_N_4_ and MoS_2_ simultaneously without changing their phase structure.

**Figure 3.**
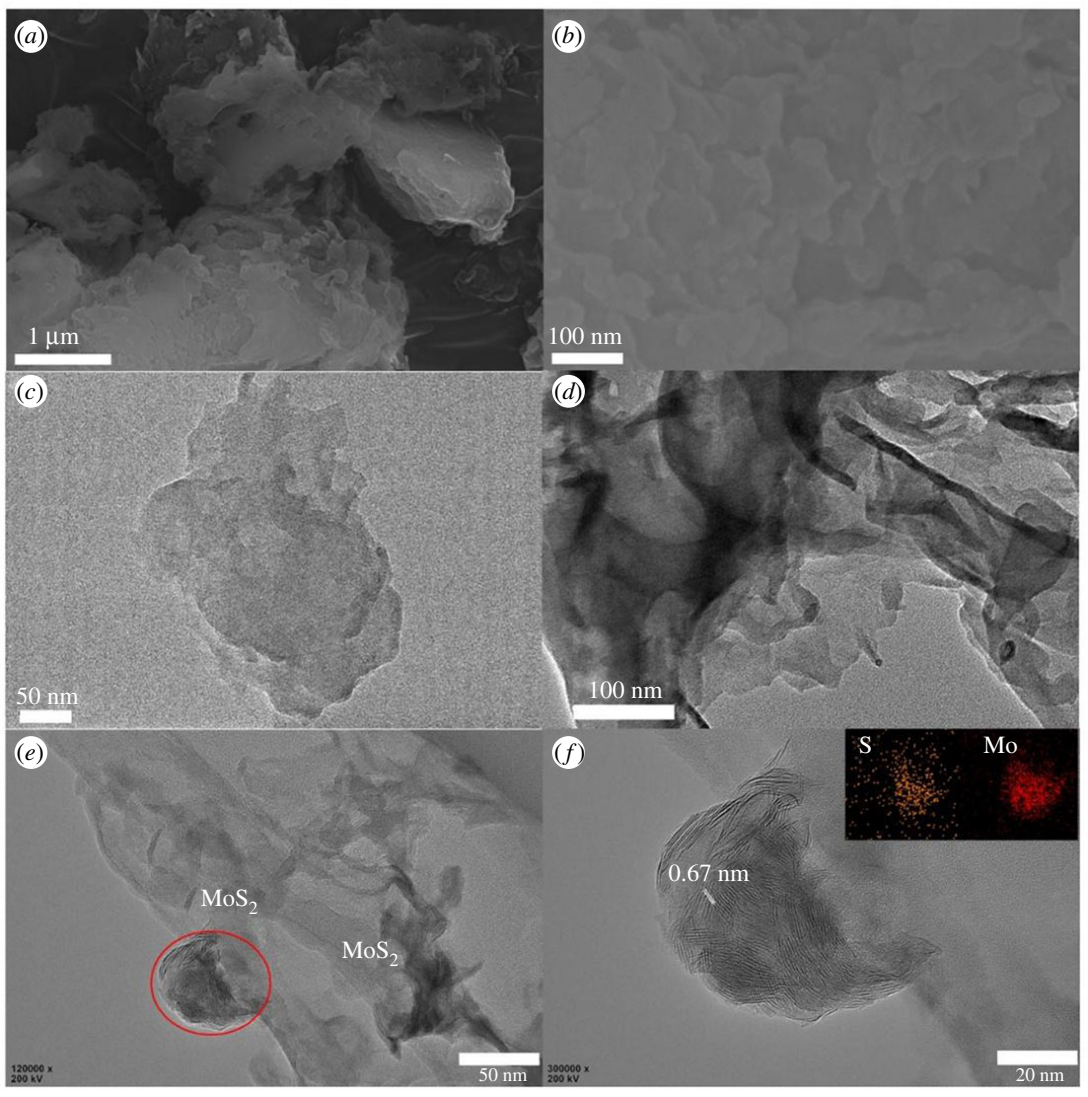
(*a,b*) SEM images of g-C_3_N_4_ and 1.25% MoS_2_. (*c,d*) TEM images of g-C_3_N_4_. (*e*) TEM image of 1.25% MoS_2_. (*f*) Magnified image of the area circled in red in (*c*). Inset of (*f*) is the corresponding elemental mapping of Mo and S elements.

The morphology and microstructure under SEM and TEM are shown in figure 3. The samples with MoS_2_ retained the stacked lamellar particle structure typically as did pure g-C_3_N_4_ [13]. The layered structure can be clearly seen from figure 3*b* with the average lateral size of several hundred nanometres and irregular edges. Compared with the layered amorphous structure of pristine g-C_3_N_4_ displayed in figure 3*c*,*d*, that of the composite material showed well-crystalized parts in figure 3*e*, which can be identified as the MoS_2_ layer. Figure 3*f* displays a magnified image of the area circled in red in (*e*). The elemental mapping confirmed the existence of Mo and S elements in these nanosheets. The crystal lattice can be determined from the high-resolution TEM of 0.67 nm, typically as the (002) plane of MoS_2_. In short, the TEM images, in accordance with XRD, showed a clear MoS_2_ lattice structure in the g-C_3_N_4_ framework, indicating that high-quality MoS_2_ nanolayers were well distributed to form the two-dimensional/two-dimensional heterojunction with g-C_3_N_4_. This structure is very promising for photocatalytic reactions.

**Figure 4.**
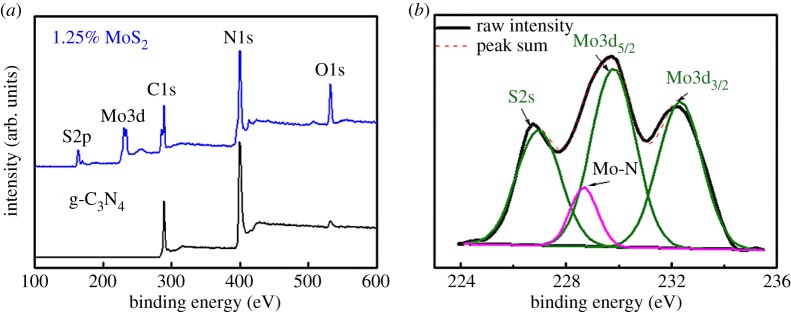
XPS spectra of: (*a*) a survey spectrum of pristine g-C_3_N_4_ and a sample of 1.25% MoS_2._ (*b*) High resolution of peak Mo3d from the sample of 1.25% MoS_2_.

The surface chemical information, including the surface elemental ratio and valence states of Mo, was investigated by XPS. Table 1 presents the Mo and S ratios obtained from the XPS analysis. The ratios of Mo and S are near to 2 : 1, which indicates the chemical composition of MoS_2_. Figure 4*b* depicts the Mo 3d high-resolution spectra of a sample of 1.25% MoS_2_ as an example. After peak fitting, two distinct peaks centred at 229.4 eV and 232.2 eV were observed and ascribed to Mo 3d_5/2_ and Mo 3d_3/2_ doublets of MoS_2_, respectively. The first peak, centred at 227.1 eV, agrees well with that of the 2s binding energy of elemental S [57]. More importantly, the peak centred at 228.6 eV was determined as the Mo─N bonding [58], suggesting a strong interlayer effect between MoS_2_ and g-C_3_N_4_. On the basis of the above results and discussion, it can be concluded that MoS_2_ can be successfully loaded onto g-C_3_N_4_ to form a proper crystal structure and chemical composition.

**Table 1.** Surface chemical compositions, specific surface areas, reaction constant for RhB decomposition, and the calculated reaction constant per unit area of prepared samples.

sample	g-C_3_N_4_	0.5% MoS_2_	1.25% MoS_2_	2.5% MoS_2_
Mo/N %	—	0.8	2.4	5.6
S/N %	—	1.8	4.3	10.0
SSA (m^2^ g^−1^)	11	25	45	93
react. const. (h^−1^)	0.26	0.93	1.53	1.13
react. const. per unit	0.024	0.037	0.034	0.012

**Figure 5.**
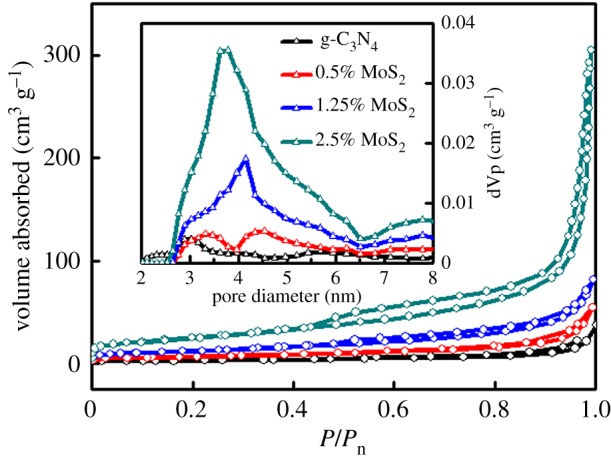
N_2_ adsorption/desorption isotherms; inset: pore size distribution (PSD).

The porous structures of the materials were measured by N_2_ adsorption–desorption at 77 K as shown in figure 5. All the samples exhibited a hybrid of a II- and IV-type isotherm with a hysteresis loop at relative pressures (*P*/*P*_0_) ranging from 0.5 to 1.0, implying the existence of mesopores (2–50 nm). The SSA can be further calculated using the BET method. The SSA, summarized in table 1, dramatically increased with the increase of MoS_2_ contents. The PSD of MoS_2_-modified samples plotted in the inset figure shows a broad peak in the range 3–6 nm, while the pure g-C_3_N_4_ displayed negligible mesoporous structure. The porous structure suggests that the *in situ* modification of MoS_2_ is expected to enhance the photocatalytic activity by exposing more surface photocatalytic active sites [59,60].

**Figure 6.**
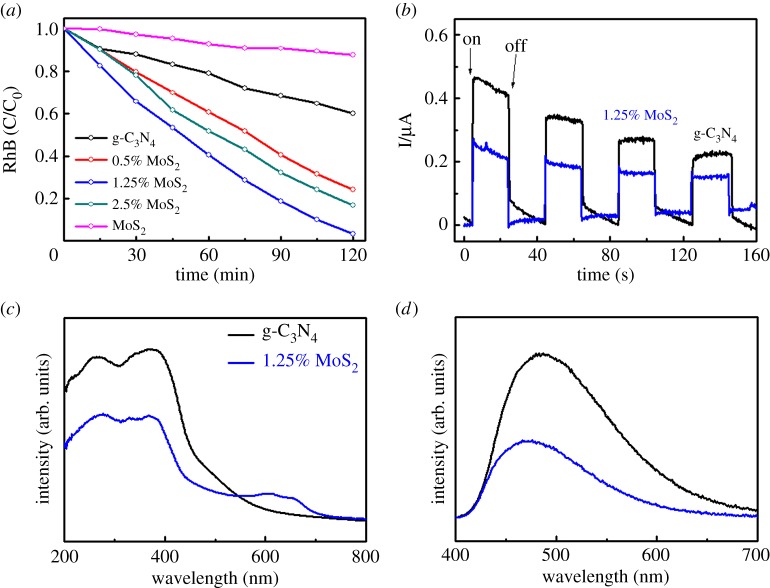
(*a*) Photocatalytic degradation of RhB. (*b*) Transient photocurrent responses. (*c*) UV–vis DRS spectra of all samples and (*d*) PL spectra of all samples.

In this work, photocatalytic activity is measured by RhB degradation in water. Figure 6*a* illustrates the concentration changes of RhB in water that was degraded under visible light by various photocatalysts. Under the same conditions, g-C_3_N_4_/MoS_2_ displayed remarkably improved photocatalytic activity compared with pure g-C_3_N_4_ or pure MoS_2_. The reaction constants for the concentration changes of RhB are also summarized in table 1. Among the three modified samples, 1.25% MoS_2_ possesses the highest reaction constant of 1.53 h^−1^, which is nearly six times the activity of pristine g-C_3_N_4_. To investigate the effect of different SSAs and porosity, the reaction constant per unit area was then calculated by dividing the reaction constants by SSA separately. The samples 0.5% MoS_2_ and 1.25% MoS_2_ showed similarly higher activity per unit area than g-C_3_N_4_. However, too much MoS_2_ resulted in a lower reaction constant per unit area, as shown when sample 2.5% MoS_2_ is compared with pure g-C_3_N_4_.

**Figure 7.**
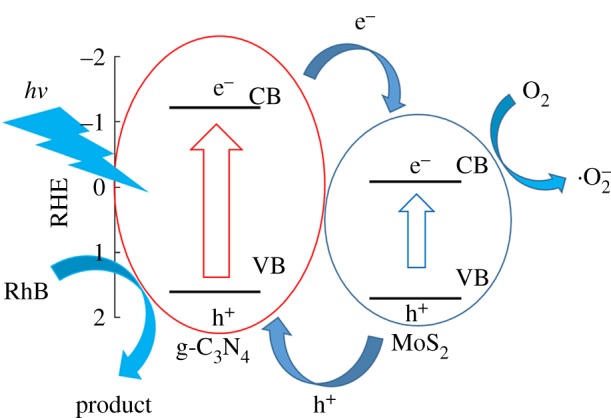
Schematic illustration of charge carrier transformation in the g-C_3_N_4_/MoS_2_ heterojunction.

Enhanced photoactivity is ascribed to extended light absorbance at higher wavelengths and the timely separation of photo-generated charges, as confirmed by the UV–vis DRS and PL emission spectrum results. As illustrated in figure 6*c*, the main absorption edge of all the samples at approximately 440 nm remains unchanged regardless of the MoS_2_ concentration, corresponding to the bandgap of 2.8 eV of g-C_3_N_4_. No shift of the absorption wavelength edge demonstrates MoS_2_ forming the heterojunction structure on the surface of g-C_3_N_4_ nanolayers. The introduction of MoS_2_ to form the heterojunction contributes to enhanced absorption in the range 570–690 nm, providing the possibility of an increased visible light response. The PL intensity, which is indicative of the recombination of photo-generated carriers, is notably suppressed by MoS_2_ heterojunctions. The efficiently separated photo-induced electrons and holes will greatly contribute to the photocatalytic reaction. As depicted in figure 7, photo-generated electrons transferred from g-C_3_N_4_ to the conduction band of the surface MoS_2_. Owing to the trapping effect of MoS_2_, electrons are trapped on MoS_2_, while holes move to g-C_3_N_4_ to enhance the separation of photo-generated carriers.

To further investigate the photoelectronic process under illumination the photocurrent was measured both on pristine C_3_N_4_ and on the C_3_N_4_/MoS_2_ composites. The sample 1.25% MoS_2_ generated a lower photocurrent, which demonstrates significant electron transfer from the C_3_N_4_ layer to the surface MoS_2_ layer with an electron trapping effect to help carrier separation [61,62]. It should be noted that the gradual decrease of photocurrent was due to the slow detachment of sample from the FTO surface [61].

However, too much MoS_2_, as sample 2.5% MoS_2_, resulted in a lower reaction constant per unit area, i.e. half of that of g-C_3_N_4_. This may be ascribed to the shielding effect of the higher amount of MoS_2_ on the g-C_3_N_4_ layers at low wavelengths to inhibit the generation of photo-carriers. This phenomenon might be explained in two ways [50]: the greater amount of MoS_2_ covers some active sites on the g-C_3_N_4_ surface; or the surface MoS_2_ absorbs too much light to suppress the light absorption of g-C_3_N_4._ In this paper, we found that the optimum ratio of MoS_2_ is approximately 1.25%, considering the overall performance of the catalyst.

**Figure 8.**
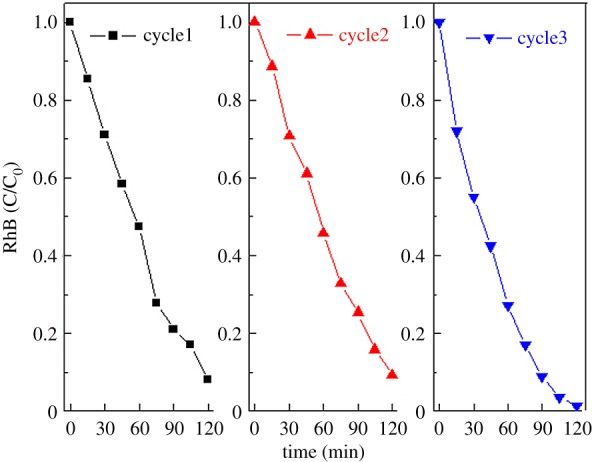
Repeated catalytic measurement of sample 1.25% MoS_2_ for RhB degradation under visible light irradiation.

The stability of the catalysts was confirmed by repeated cycling measurements. As shown in figure 8, after three cycles, there was no considerable decrease in photocatalytic efficiency, indicating the stability of the as-synthesized g-C_3_N_4_/MoS_2_ heterojunction catalysts.

## 4. Conclusion

In summary, a heterostructural g-C_3_N_4_/MoS_2_ composite was fabricated by a one-step heating procedure. Both g-C_3_N_4_ and MoS_2_ have nanosheet-like structures with sufficient contact interfaces to form a heterojunction. The mesoporous structure of g-C_3_N_4_ can be tuned by different amounts of MoS_2_. The incorporation of MoS_2_ contributes a lot to the efficient charge separation, resulting in a significantly enhanced photocatalytic performance and increased photocurrent. However, an excessive amount of MoS_2_ may also decrease the photocatalytic activity of g-C_3_N_4_. Particularly, a loading of 1.25% MoS_2_ to the g-C_3_N_4_ is demonstrated to show the best photocatalytic performance.

## Supplementary Material

Supporting Information

## Data accessibility

SEM, PL and UV–vis data of other samples, as well as the absorption spectrum of RhB, are available in the electronic supplementary material.
