# Supplementary material for: Synthesis and photocatalytic activity of mesoporous g-C3N4/MoS2 hybrid catalysts
Source: R Soc Open Sci. 2018 May 16;5(5):180187. doi: 10.1098/rsos.180187 (PMC5990742; doi:10.1098/rsos.180187)
Supplement: Supporting Information [file rsos180187supp1.docx]

Supporting Information


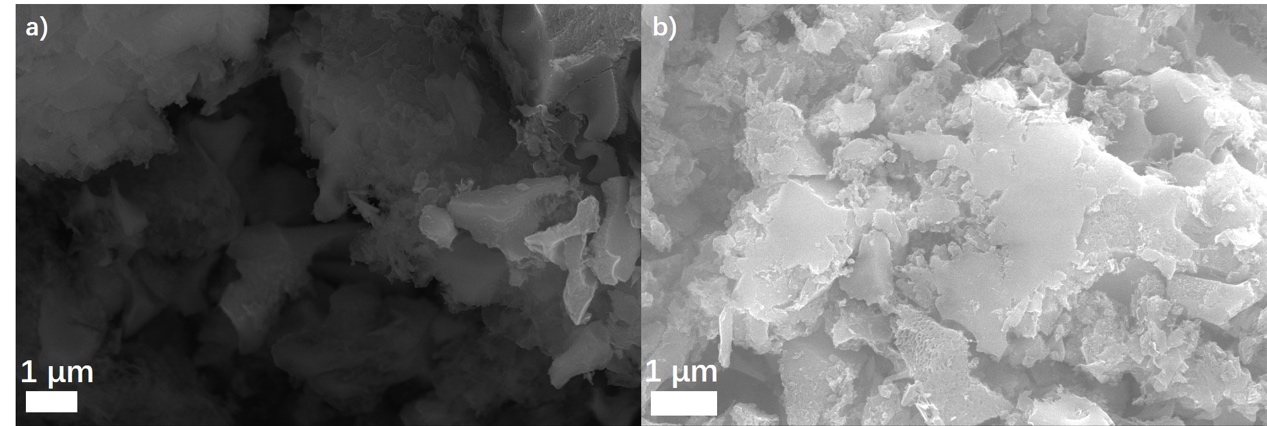


Figure S1 SEM images of a) 0.5 % MoS_2_/g-C_3_N_4_ and b) 2.5 % MoS_2_/g-C_3_N_4_


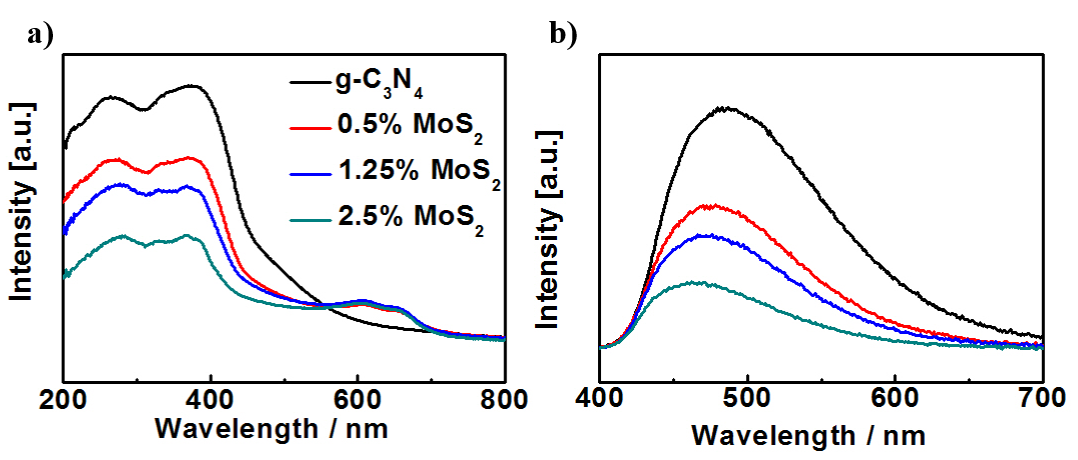


Figure S2 (a) UV-vis DRS spectra of all samples; (b) PL spectra of all samples.


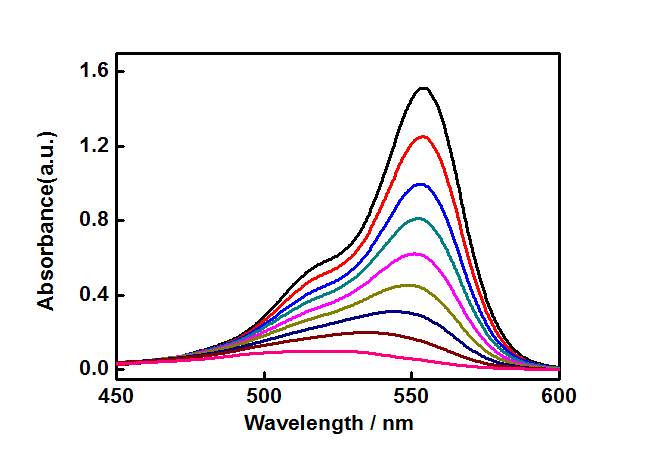


Figure S3 Absorption spectrum of RhB solution during photocatalytic measurement by sample 1.25 % MoS_2_.
